# Student Perspectives on a Smoothie-Based Educational Program Designed Using Social Cognitive Theory and Choice Architecture

**DOI:** 10.3390/ijerph23030359

**Published:** 2026-03-12

**Authors:** Amelia Sullivan, Bryn Kubinsky, Emma Watras, Kathyrn Yerxa, Kayla Gayer, Elizabeth Hufnagel, Kathleen A. Savoie, Jade McNamara

**Affiliations:** School of Food and Agriculture, University of Maine, Orono, ME 04469, USA; amelia.sullivan@maine.edu (A.S.); bryn.kubinsky@maine.edu (B.K.); emma.watras@maine.edu (E.W.); kate.yerxa@maine.edu (K.Y.); kayla.gayer@maine.edu (K.G.); elizabeth.hufnagel@maine.edu (E.H.); ksavoie@maine.edu (K.A.S.)

**Keywords:** Social Cognitive Theory, Choice Architecture, adolescents, nutrition security, fruit consumption, rural, nutrition programming, process evaluations, qualitative research, mixed-methods research, smoothies, frozen fruit

## Abstract

**Highlights:**

**Public health relevance—How does this work relate to a public health issue?**

- Rural adolescents often face barriers to nutrition security, and schools are a primary setting where nutrition programs can reach students equitably.
- This study evaluates a theory-based smoothie and nutrition education program during the school lunch period.

**Public health significance—Why is this work of significance to public health?**

- Students reported high likeability across all program sessions, with strong willingness to consume smoothies again at school lunch.
- Student feedback reflected key mechanisms of behavior change and environmental influences consistent with Social Cognitive Theory and Choice Architecture.

**Public health implications—What are the key implications or messages for practitioners, policy makers and/or researchers in public health?**

- Brief, point-of-service nutrition education paired with experiential tastings may be a feasible approach for supporting nutrition knowledge and engagement within existing school meal systems.
- Implementation of such programming can be strengthened by incorporating more hands-on and peer-based components.

**Abstract:**

**Background/Objective:** Helping Early Adolescents Live Their Healthiest Youth (HEALTHY) is a four-session, smoothie-based nutrition education program grounded in Social Cognitive Theory (SCT) and Choice Architecture, designed to promote nutrition security among rural adolescents. This study examined students’ experiences with the program, including perceptions of likeability and perceived learning, as well as the theoretical mechanisms shaping engagement. **Methods:** A mixed-methods evaluation was conducted in two rural middle schools where the programming was delivered. Process indicators were assessed using brief paper-based surveys administered after each program session. Quantitative items captured likability and willingness to consume smoothies again (at home or school lunch), and qualitative open-ended responses were analyzed inductively. Post-program focus groups were conducted with a subsample of participants (*N* = 18) and analyzed deductively using a coding framework aligned with SCT constructs. **Results:** Across sessions, students (*N* = 360) reported high smoothie likeability, with fewer than 15% indicating dislike of any recipe. Willingness to consume smoothies again remained high, with affirmative responses ranging from 72% to 94% at home and 79% to 97% at school lunch. Inductive thematic analysis indicated that 53% of survey responses reflected session-aligned nutrition knowledge, along with themes related to acceptability and suggestions for improvement. Focus group findings reflected multiple SCT constructs, including knowledge awareness, self-efficacy, and goal-setting, as well as environmental influences regarding engagement, consistent with Choice Architecture. **Conclusions:** Findings indicate that the HEALTHY program was well received by rural adolescents and reflected key theoretical mechanisms underlying its design. Student feedback guides future program refinement.

## 1. Introduction

Understanding how adolescents experience school-based nutrition programming is essential for determining whether programs are feasible, acceptable, and capable of producing meaningful behavior change in real-world settings [1,2]. While summative evaluations provide valuable objective insights, they often do not capture the mechanisms that shape engagement or explain why programs may succeed or fail [3]. As a result, evaluations that center on participant experiences are critical for interpreting program effectiveness and informing refinement, sustainability, and scale-up.

Process evaluations play an essential role in this work by examining how participants interact with program components and by identifying contextual factors that influence implementation and sustainability [3,4]. Within the school food environment, time constraints, social dynamics, and competing food options strongly influence student decision-making [5,6,7]. However, student perceptions, learning, and agency also represent meaningful outcomes, particularly when programs are designed to influence motivation, confidence, and engagement [1,2]. Together, process and student-centered outcome evaluations provide complementary insights into how and why school-based nutrition programs function beyond what can be captured through summative objective measures alone.

Among the student-centered outcomes of interest in school-based nutrition programming is the development of nutrition knowledge [8,9]. Strengthening nutrition knowledge during early adolescence may support informed diet-related choices. This is particularly important in structured eating environments, such as the school food environment, where daily food choices are made [2,6,10]. Nutrition knowledge reflects understanding and recall of nutrition-related concepts delivered through educational activities. In rural settings, where structural barriers may shape dietary behaviors and limit access to health-promoting resources [11,12,13], evaluating adolescents’ nutrition knowledge may provide meaningful insight into how effectively school-based nutrition education programs are received and understood.

Behavioral theories offer a structured framework for examining how such knowledge may develop and which mechanisms within multicomponent programming are most influential [14]. Prior school-based research suggests that combining theory-driven education with environmental modifications may strengthen both motivation and opportunities for healthier choices [15,16]. Accordingly, Social Cognitive Theory (SCT) and Choice Architecture provide a complementary, dual-level lens for both developing and evaluating nutrition programs within the complex social and physical context of the school food environment. Broadly, SCT emphasizes the interpersonal and cognitive processes that influence behavior, while Choice Architecture focuses on how environmental conditions shape everyday decision-making [17,18]. When applied together, these frameworks allow for the evaluation of how individual learning and social interaction intersect with environmental cues to influence engagement. 

Social Cognitive Theory conceptualizes behavior change as the dynamic, mediating interaction among cognitive, behavioral, and environmental influences, a process described as reciprocal determinism [17]. Key constructs of the SCT include behavioral capability, perceived health benefits, observational learning, goal-setting, self-efficacy, self-agency, and self-regulation [17]. In school-based nutrition programs, SCT-informed approaches have been associated with improvements in adolescent diet quality, increased willingness to try new foods, and greater confidence in applying nutrition knowledge in daily life [19,20,21,22,23,24]. Importantly, SCT also highlights the role of social models and interpersonal interactions, suggesting that educators, peers, and facilitators can actively shape students’ learning and motivation [17].

Complementary to SCT, Choice Architecture, a framework rooted in Behavioral Economics, focuses on how environmental cues influence decision-making [18,25,26]. Choice Architecture strategies seek to ‘nudge’ individuals towards healthier behaviors by restructuring how information and options are presented to increase visibility, convenience, and appeal, or by providing timely cues that reinforce learning and support self-regulation. Within school meal programs, these strategies can influence food selection and consumption by making healthier options easier to choose or more automatic [18,25,26,27,28,29,30,31]. While often described as structural, Choice Architecture in practice is also shaped by social cues, norms, and interactions within food environments, particularly among adolescents.

Student perspectives are, therefore, central to evaluating whether theoretical mechanisms operate as intended within programming [1,2,32]. Adolescents can offer their critical insights into what they learned, how they experienced program activities, and which components feel engaging, relevant, or feasible. Their perspectives can also highlight how social interactions, environmental cues, and opportunities for agency shape participation and decision-making. Furthermore, expanding the evidence on student-centered experiences with nutrition programming is particularly important in rural settings, where disparities in food access, cafeteria resources, and nutrition security may influence how programs are received [12,33,34,35].

The purpose of this study was to conduct a mixed-methods evaluation of HEALTHY (Helping Early Adolescents Live Their Healthiest Youth), an educational, four-session, smoothie-based program grounded in SCT and Choice Architecture, designed to promote nutrition security among rural adolescents. Specifically, this study aimed to assess: (1) student-reported likability and willingness to consume smoothies again; (2) student-reported nutrition knowledge immediately following participation; and (3) the extent to which student reflections aligned with theoretical constructs embedded in the program design.

We hypothesized that students would report positive experiences and recall nutrition knowledge from session-specific educational content, and that their post-session reflections would align with the core constructs of SCT and Choice Architecture, providing insight into the mechanisms by which the program influenced engagement within the school food environment.

## 2. Materials and Methods

### 2.1. Study Design

This study employed a mixed-methods evaluation design to examine program implementation indicators and student-centered outcomes associated with the HEALTHY program, using brief post-session surveys and post-program focus groups to explore students’ perceptions, learning experiences, and engagement. Together, these approaches were selected to capture how students experienced the program and to identify theoretical mechanisms shaping engagement beyond what can be assessed through summative objective outcomes alone. The University of Maine Institutional Review Board approved this study (#12-22-2024, 22 December 2024).

### 2.2. Program Description and Theoretical Framework 

Helping Early Adolescents Live Their Healthiest Youth (HEALTHY) is a nutrition education program based on frozen fruit smoothies designed to promote nutrition security among rural adolescents. The program was implemented during regularly scheduled lunch periods from February to April 2025 at two rural middle schools serving students in grades three through eight [36].

The program consisted of four bi-weekly sessions. During each session, students were offered an eight-ounce, nutrient-dense smoothie that met National School Lunch Program requirements and was paired with brief, facilitator-led nutrition education. Sessions were delivered at a designated program table within the cafeteria and facilitated by trained research assistants who were Registered Dietitian Nutritionists and/or senior undergraduate dietetic students [36]. All students present during the regularly scheduled lunch period were invited to participate, although participation was voluntary and varied depending on student attendance and interest. The program was provided to schools at no cost.

Program design and implementation were guided by SCT and Choice Architecture. Social Cognitive Theory served as the primary behavioral framework, informing both the educational content and facilitator–student interactions. Sessions emphasized experiential tasting, observational learning, self-efficacy building, and student agency. Research assistants acted as intentional social models, delivering brief verbal and visual education, facilitating discussion, and guiding structured SMART (Specific, Measurable, Actionable, Relevant, Time-Bound) goal-setting activities. Students were prompted in subsequent sessions to reflect on previously established goals, reinforcing self-regulation and accountability. Small fruit-themed incentives were provided following the goal-setting activities to support engagement.

Choice Architecture informed the physical and social design within the school food environment. Environmental strategies included positioning the program table in a highly visible location, using colorful fruit-themed table coverings, displaying visually engaging educational materials, serving smoothies in clear cups with bright, colorful straws, and minimizing the time and effort required to participate. Social cues were intentionally embedded through repeated exposure, positive peer interactions, and facilitator encouragement to reinforce norms around fruit consumption and program engagement. Locating the program table within the shared cafeteria space allowed students to observe peers participating, discuss flavor preferences, and engage in brief facilitator-guided education during tasting. Together, SCT and Choice Architecture shaped an environment in which the program’s educational messaging, social interactions, and environmental cues could synergistically influence student experiences and decision-making.

Each of the four sessions featured a distinct smoothie and an aligned nutrition education theme. Session one introduced the “Power Play Shake,” accompanied by nutrition education focused on fueling for sports performance, muscle recovery, and sustainable energy. Session two featured the “Citrus Splash,” emphasizing immunity and skin health. Session three highlighted the “Hydration Hero,” focusing on natural hydration and reducing intake of sugar-sweetened beverages. Session four concluded with the “Maine Maple Blueberry,” which centered on local and seasonal food choices. Across sessions, educational messaging intentionally connected the featured ingredients to key nutrients and their functional roles in the body. Additional details regarding program development, implementation logistics, and summative objective outcomes have been reported elsewhere [36].

### 2.3. Participants and Recruitment

All students who participated in the HEALTHY program were invited to complete the brief paper-based post-session surveys. Eligible participants were students enrolled in grades three through eight at the two experimental program schools. Surveys were administered immediately following each program session during the school lunch period. Students were eligible to participate in each session regardless of prior smoothie consumption and/or attendance at previous sessions.

As part of the program protocol, parental consent was obtained prior to data collection. Schools distributed the parental consent letter through school newsletters and electronic communication platforms. Parents provided electronic consent via Qualtrics, and trained research assistants obtained verbal assent from students on data collection days prior to survey completion.

Focus group participants were drawn from this same pool of program participants. A subsample of students was invited to participate in focus group discussions to provide more in-depth perspectives on their experiences with the HEALTHY program using a convenience sampling approach. Recruitment information was distributed through school newsletters and electronic communication platforms and coordinated by the school principals. For focus group participation, parents provided written consent, which was collected on the day of the discussion, and students provided verbal assent before participating.

### 2.4. Instruments, Measures, and Procedures 

#### 2.4.1. Post-Session Student Surveys

Student experiences with the HEALTHY program were assessed using a brief paper-based survey developed by the lead researchers. The survey consisted of six items and was administered immediately following each program session during the school lunch period. After visiting the program table to receive their smoothie and brief nutrition education, students were provided the survey and instructed to complete it at their convenience before the lunch period ended. The timing was intended to capture immediate reactions to both the smoothie and the educational content while minimizing disruptions to the lunch schedule. All responses were anonymous and required approximately one to two minutes to complete.

The first survey item prompted students to identify which smoothie they had tried (e.g., session one, session two, session three, or session four’s recipe). Smoothie likability was assessed using the question: “How much did you like the taste of this smoothie?” with response options of “Loved it,” “It was good,” “It was okay,” and “Didn’t like it much.”

Two items assessed students’ willingness to consume the smoothie again in different contexts: (1) “Do you think you would try making or drinking a smoothie like this at home?” and “Would you drink this smoothie again if offered at lunch?” Response options for both items included “Yes,” “Maybe,” and “Probably not.”

Two open-ended items assessed perceived learning and suggestions for program improvement: (1) “What was one thing you learned about today’s smoothie and education activity?” and (2) “What could make this activity more fun or interesting for you?”

To mitigate potential bias related to hypothesized positive outcomes, survey responses were collected anonymously, and students were informed that participation would not affect their access to the HEALTHY programming. Open-ended items were intentionally phrased neutrally and included prompts for both positive feedback and suggested improvements. No demographic identifiers (e.g., age, grade level, gender) were collected as part of this evaluation.

#### 2.4.2. Student Focus Groups

Focus groups were conducted with a convenience subsample of students who participated in the HEALTHY program to gather in-depth perspectives on program experiences, educational messages, and the cafeteria environment. Approximately 77 students participated in the HEALTHY programming across the two experimental schools. We aimed to recruit at least 20% of participants for focus groups (minimum *n* = 16) [37]. Ultimately, 18 students across grades four through eight participated, representing approximately 23% of the eligible sample. Students were organized into three focus groups of five to seven participants, with two groups held at one experimental school and one at the second.

Focus groups were held in quiet spaces within the school during non-instructional time, approximately one week after the program concluded. Each discussion was facilitated by one of two trained moderators with more than 10 years of collective experience in qualitative research and supported by at least two note takers. All focus groups were audio recorded for accuracy using an iPad and lasted approximately 28 min.

A semi-structured focus group guide was used to prompt discussion of students’ perceptions of the smoothie tasting sessions, the educational messages, and the school cafeteria environment. See Table 1 for the complete focus group guide. 

### 2.5. Data Analysis

Data analysis was guided by the study’s mixed-methods evaluation framework and theoretical underpinnings. Descriptive quantitative analyses were used to summarize process indicators captured through the surveys, while qualitative analyses were used to explore student-centered outcomes and theoretical mechanisms shaping engagement. Quantitative and qualitative data addressed distinct but complementary evaluation aims and were intentionally integrated during interpretation to provide a more comprehensive understanding of program implementation and student experiences.

Quantitative items from the paper-based post-session surveys were summarized descriptively using IBM SPSS Statistics for Mac (v29.02.0; IBM Corp., Armonk, NY, USA). Smoothie likability responses were reported as frequencies for each response option across program sessions. For the two items assessing students’ willingness to consume smoothies again at home and at school lunch, responses were grouped into affirmative (“Yes” and “Maybe”) and negative (“Probably not”) categories to facilitate interpretation of overall likeability across sessions. Because participation was anonymous and voluntary, this data is interpreted at the group level.

#### 2.5.1. Inductive Thematic Analysis of Open-Ended Survey Responses

Open-ended survey responses assessing student learning and suggestions for program improvement were analyzed using inductive thematic analysis, following established qualitative methods to minimize confirmation bias [37,38]. First, one research assistant entered all paper-based responses in Microsoft Excel (Version 16., Microsoft Corporation, Redmond, WA, USA). Three independent coders then read all responses to familiarize themselves with the data. The coders then independently created emerging themes. Using an iterative consensus process, the coders subsequently met to compare themes, resolve any discrepancies, and reach agreement on the final major themes. Theme saturation was assessed based on the frequency and repetition of student responses across themes [37,38]. Furthermore, the proportion of students contributing to each major theme was calculated to provide descriptive context in interpreting qualitative themes. Excerpts are labeled by session number for survey responses (e.g., “session 1 student” and so forth).

#### 2.5.2. Deductive Thematic Analysis of Student Focus Groups

Student focus group discussions were analyzed deductively using a coding framework aligned with the domains and constructs of the SCT [17,37,38,39]. Audio recordings were transcribed verbatim into Microsoft Word documents (Version 16., Microsoft Corporation, Redmond, WA, USA) by a research assistant and cross-verified for accuracy by two additional research assistants. Three independent coders reviewed the transcripts and then applied the a priori codebook, identifying text segments that reflected each theoretical construct and organizing them within a Microsoft Excel spreadsheet (Version 16., Microsoft Corporation, Redmond, WA, USA). See Table 2 for the codebook used by coders.

Coders met to compare interpretations and reach agreement on the proper text segments for each a priori code [37,38,39]. The number of students contributing to each a priori code was tallied to provide descriptive context and assess saturation. Emergent themes that did not align with the a priori SCT framework were documented and are reported alongside the deductive findings. Excerpts are labeled by school sites (e.g., “school A student” and “school B student”).

## 3. Results

### 3.1. Post-Session Survey Findings 

Paper-based post-session surveys were used to assess student-reported smoothie likeability, willingness to consume smoothies again, and perceived learning following each HEALTHY program session. Across the four sessions, a total of 360 surveys were completed (session one: *n* = 100; session two: *n* = 88; session three: *n* = 89; and session four: *n* = 83).

Overall, students reported high likeability of the smoothies across all sessions. When combining the response options “Loved it” and “It was good,” the session one smoothie received the highest overall likeability rating (87%, *n* = 97), followed by the session two smoothie (81.9%, *n* = 72). Session four’s smoothie received the lowest likeability rating, with 15.7% (*n* = 13) of students reporting that they “Didn’t like it much.”

Students’ willingness to consume smoothies again at school and at home was above 72% across all four sessions. Specifically, affirmative responses for willingness to consume or make smoothies again at home ranged from 72.3% to 94.0%. In contrast, affirmative responses for willingness to consume smoothies again at school ranged from 79.5% to 97.0%. Frequencies of student-reported likeability level and willingness to consume again at home and at school across program sessions are summarized in Table 3.

Inductive thematic analysis of open-ended survey responses revealed four major themes: (1) session-aligned nutrition knowledge, (2) HEALTHY program acceptability, (3) program improvement ideas, and (4) unclear knowledge or improvement ideas.

Over half of the participating students (53.0%, *n* = 191) demonstrated session-aligned nutrition knowledge (theme one), reflecting immediate recall and articulation of session-specific educational concepts post-sessions. Students frequently referenced nutrients, ingredient functions, and health-related messages aligned with each session’s focus. For example, one student shared, “Milk is good for your bones, and if we had smoothies in the morning, it would fuel us” (session 1 student).

Nearly one-third of students (29.0%, *n* = 106) expressed HEALTHY program acceptability (theme two), describing enjoyment of the program and satisfaction with the activities. Representative responses include “I like your visits” (session 3 student) and “I think it’s great” (session 1 student).

Program improvement ideas (theme three) were identified among 38.0% (*n* = 139) of students. Suggestions commonly reflected their interest in additional smoothie offerings, recipe modifications, and increased opportunities for hands-on engagement. Examples included requests for “A combination of more smoothie flavors!” (session 2 student) and experiential elements such as “If I got to help make it” (session 3 student).

Finally, unclear knowledge or ideas for improvement (theme four) were observed among 23.0% (*n* = 83) of students, who indicated uncertainty about what they learned or how the program could be improved. Responses such as “I don’t know” and “?” were common within this theme. Additional representative quotes for each theme are presented in Table 4.

### 3.2. Student Focus Group Findings

A subsample of students who participated in the HEALTHY program participated in focus group discussions (*N* = 18), including fourth graders (*n* = 4), fifth graders (*n* = 3), sixth graders (*n* = 4), seventh graders (*n* = 3), and eighth graders (*n* = 4).

Focus group data were analyzed deductively using a priori codes aligned with the three SCT domains (cognitive, behavioral, environmental) and respective constructs [17]. Themes and quotes are described below. Additional representative quotes are presented in Table 5.

#### 3.2.1. Cognitive Domain

Within the cognitive domain, two a priori codes were identified: (1) nutrition knowledge awareness and (2) perceived health benefits. 

Nearly half of the students (44.4%, *n* = 8) self-reported nutrition knowledge awareness, citing awareness of ingredients, nutrients, and how foods support bodily functions. For example, one student noted:

“It was really nice to figure out, […] what kind of stuff I was actually eating and what kind of stuff it was giving me, um, for nutrition.”

(school A student)

Perceived health benefits were reported by one-third of students (33.3%, *n* = 6), who described benefits such as improved energy, athletic performance, and overall well-being. One student shared:

“I really like the smoothies, and I think they are a great way how to teach other people and kids how to eat healthy and make them wanna eat healthy.”

(school B student)

The a priori code outcome expectations did not emerge during focus group discussions. However, an emergent theme, student-driven suggestions, was identified within the cognitive domain. This theme reflected students’ interest in additional learning activities and opportunities for engagement, such as incorporating games or interactive components in the sessions. For example, one student stated:

“I feel like it would be fun if we did like, some game or something.”

(school A student)

#### 3.2.2. Behavioral Domain

Three a priori codes emerged within the behavioral domain: (1) positive behavior change, (2) self-efficacy and confidence, and (3) goal-setting and self-regulation.

One-third of students (33.3%, *n* = 6) reported positive behavior change, including increased fruit and vegetable consumption and the preparation of smoothies at home. As one student explained:

“I started eating more healthy, and I started eating more healthy meals at dinner and lunch, at home on the weekends and stuff, and I started helping my mom in our greenhouse to pick more stuff and make more salads on my own.”

(school B student)

Self-efficacy and confidence were expressed by 38.3% (*n* = 7) of students, who described feeling capable of preparing smoothies independently and making healthier choices. One student stated:

“When you guys first introduced the smoothies, I tried one. The first one was really good. Ever since you kept coming back, every smoothie I’ve made at my house because they tasted really good.”

(school B student)

Goal-setting and self-regulation were described by 44.4% (*n* = 8) of students. Students characterized SMART goal-setting as achievable and motivating, noting that meeting short-term goals felt rewarding. As one student described:

“I thought they were kind of fun because you got to, you’d set a goal, and then you would meet that goal, and it would be like kind of like, ‘oh, I just met a goal and it was, only took like a week or two.’”

(school A student)

The a priori codes, observational learning, and sharing or applying nutrition knowledge did not explicitly emerge during discussions.

#### 3.2.3. Environmental Domain

Within the environmental domain, five a priori codes were identified: (1) group norms and collective efficacy, (2) interpersonal encouragement, (3) incentive motivation, (4) environmental barriers, and (5) accessibility of healthy foods.

Group norms and collective efficacy were frequently discussed and described by 55.5% (*n* = 10) of students. Students emphasized smoothie days as a shared and enjoyable experience in the cafeteria, often noting increased excitement and anticipation. One student shared:

“This has been one of the funnest years because of the smoothies, like, because sometimes we don’t know and we see you guys, and then we get all excited.”

(school B student)

Interpersonal encouragement was described by approximately one-third of students (33.8%, *n* = 7) and included support from peers, family members, and cafeteria staff. For example, one student stated:

“But if they, if their parents encourage them to eat, um, these foods, and ingest them, and drink things, not just smoothies, but drink things to help their immune system or make them as stronger athletes, or not just athletes. Uh, yeah, just to like to help them, their brain, I don’t know the words. Focus better on school and work better.”

(school A student)

Incentive motivation was discussed by 61.0% (*n* = 11) of students, who referenced visual and reward-based elements such as colorful straws and small prizes. One student noted:

“I like the color of the straws; I like how some of them are blue.”

(school B student)

Environmental barriers were identified by one-third of participants (33.3%, *n* = 6), who cited limited time for lunch, the cost of healthier foods, and the restricted availability of fresh options. As one student explained:

“Yeah, sometimes I can feel rushed because sometimes it can be too big of a lunch or sometimes it can be an itty-bitty lunch, and I’ll still be starving even though I ate everything.”

(school B student)

Accessibility of healthy foods was discussed by 55.5% (*n* = 10), with students noting that offering smoothies at school increased opportunities to try fruits they might not otherwise have access to. For example, this student states:

“I think what worked well was everyone tried it because it was there, and a lot of people tried it.”

(school A student)

The a priori code, facilitation and behavioral capability, did not emerge. However, an emergent theme, social atmosphere, was identified among 61.0% (*n* = 11) of students, capturing students’ perceptions of the lunchroom climate, peer interactions, and relationships with cafeteria staff. One student shared:

“Our lunch ladies are very nice, and they sometimes give us different options if we don’t like the lunch.”

(school B student)

## 4. Discussion

This study conducted a comprehensive mixed-methods process and student-centered outcome evaluation of the HEALTHY program through the lens of adolescent perceptions. By integrating quantitative indicators of likeability and perceived learning with qualitative insights from student reflections, this design enabled a nuanced understanding of how students experienced the program. We hypothesized that students would report positive experiences, demonstrate recall of session-specific educational content, and describe experiences aligning with the core constructs of SCT and Choice Architecture. Overall, findings indicate that the HEALTHY program was well received by students, supported immediate session-aligned nutrition knowledge recall, operationalized several SCT-informed mechanisms, and incorporated environmental nudges consistent with Choice Architecture. Together, these findings provide preliminary insight into how brief, school-based nutrition programming can meaningfully engage students, particularly in rural schools where disparities in nutrition security persist [5,6,7,12,33,34,35].

Findings from the open-ended survey items support program acceptability. Nearly one-third of students explicitly expressed satisfaction and indicated no suggestions for improvement, captured by the theme “HEALTHY program acceptability.” At the same time, some students offered constructive feedback regarding ingredient preferences, recipe modifications, and a desire for greater experiential learning. This coexistence of acceptability and specific feedback suggests active engagement rather than passive participation, underscoring the importance of incorporating student voice into program evaluation.

Across all four sessions, students reported that the smoothies served were likeable, with the majority indicating they either “Loved it” or thought “It was good.” Fewer than 15% of students reported disliking any smoothie. Willingness to consume the smoothie again at both school lunch and at home had affirmative responses exceeding 72% across all four sessions, with an average of over 90% at school lunch specifically. These findings highlight the acceptability of integrating smoothie-based offerings within the school food environment from a student perspective. Prior research in school meal settings has similarly identified smoothies as well-received [40], and these findings align with the literature, which demonstrates that taste, familiarity, and repeated exposure are key drivers of adolescents’ acceptance of healthier foods [41,42,43,44,45].

Students frequently articulated session-specific educational concepts immediately after participation, suggesting meaningful engagement with the nutrition content. Over half of the open-ended responses reflected recall of key nutrition concepts presented, including nutrient functions related to sports performance, immunity, hydration, and local food systems. For example, following the session focused on hydration and reducing intake of sugar-sweetened beverages (session three), students commonly noted that fruits contain water and can contribute to hydration. Although knowledge acquisition was not quantitatively assessed using a validated psychometric instrument, these qualitative findings provide preliminary evidence that brief, point-of-service nutrition education delivered during lunch may support immediate recall when paired with experiential tasting. Approximately one-quarter of students expressed uncertainty regarding what they learned or how the program could be improved, reflecting variability in engagement and reinforcing the value of multi-method evaluation approaches to capture student experiences. Notably, unlike many SCT-informed programs that rely on longer, classroom-based or multi-month curricula [19,20,21,22,23,24], this program was intentionally brief and embedded within the school lunch period, highlighting the feasibility of delivering theory-informed education within existing school structures.

To further understand how students experienced the program within its theoretical framing, focus group findings were examined through SCT domains. Within the cognitive domain, students described increased “nutrition knowledge awareness” and “perceived health benefits,” aligning with both educational objectives, post-session survey findings, and prior SCT-based programs/interventions that have demonstrated improvements in health-related knowledge and behaviors among adolescents (e.g., increased fruit and vegetable intake) [19,20,21,22,23,24]. While the a priori code “outcome expectations” did not explicitly emerge as a discrete construct, students frequently described feeling healthier, more energized, or better able to perform physically, suggesting implicit outcome-related beliefs consistent with SCT.

Within the behavioral domain, students reported enhanced “self-efficacy and confidence,” engagement in “goal-setting and self-regulation” through SMART goals, and perceived “positive behavior change.” The prominence of goal-setting is notable, as this component was intentionally embedded across sessions and framed as achievable and relevant by facilitators. In contrast, “observational learning” and “sharing or applying nutrition knowledge” did not emerge during focus group discussions. Although students participated alongside peers, peer modeling may not have been sufficiently emphasized. Prior research suggests that participatory and peer-led approaches can strengthen observational learning and peer/social modeling [46,47], representing a potential opportunity for future school-based nutrition programming.

The environmental domain was the most robustly represented across focus group discussions, reflecting both the cafeteria-based nature of the programming and the intentional integration of Choice Architecture strategies. Students described session days as socially engaging experiences characterized by collective excitement, supportive peer interactions, and positive relationships with facilitators. The emergence of a positive “social atmosphere” underscores the importance of lunchroom climate in shaping student engagement with nutrition programs. This finding is not surprising, as the literature shows that peer influence and the cafeteria environment are essential to adolescents’ experiences and healthy behaviors [2,32,48].

Students also identified visually appealing elements, such as colorful straws and fruit-based incentives, as motivating. These features align with Choice Architecture principles emphasizing visibility, appeal, and immediate reinforcement [18,25,26,27,28,29,30,31]. Previous studies have shown that taste tests combined with Choice Architecture strategies, including presentation and incentive-based approaches, can support healthier food selection within the school food environment [6,28,29]. At the same time, students identified environmental barriers, including limited time, cost constraints, and restricted access to fresh foods. These reflections underscore persistent structural disparities within rural communities and provide important context for interpreting both program strengths and limitations [5,6,7,12,33,34,35]. Outside of the school food environment, some students described preparing smoothies at home using program-provided recipe cards or engaging in other health-related behaviors influenced by program participation. While behavior outside the school was not measured, these findings suggest that school-based nutrition education programs may influence behaviors across settings and point to future evaluation objectives in such programming.

Overall, the positive convergence of SCT constructs and Choice Architecture strategies suggests that integrating behavioral theory with environmental design may enhance both intrinsic-level engagement and extrinsic environmental support. The prominence of environmental themes is not unexpected, given the cafeteria-based nature of the programming and the intentional design. By situating nutrition education directly within the school food environment and pairing it with appealing, accessible options, the HEALTHY program created conditions that supported exposure, engagement, and participation. Consistent with evidence supporting multicomponent approaches [15], these findings suggest that integrating behavioral theory with environmental nudges may be a promising strategy for improving nutrition security among rural adolescents within the school setting.

### Strengths and Limitations

This study has several notable strengths. First, it employed a comprehensive mixed-methods process and student-centered outcome evaluation design that intentionally integrated quantitative survey data with qualitative insights from open-ended responses and focus group discussions. This approach enabled a nuanced understanding of how students experienced the program and highlighted theoretical mechanisms shaping engagement that summative, objective-only evaluations cannot capture. The combined use of inductive and deductive thematic analysis further strengthened the analytic rigor by capturing both emergent student perspectives and theory-driven mechanisms aligned with SCT.

Second, the study prioritized student voice, an element that is often underrepresented in school-based nutrition research. By collecting immediate post-session feedback and conducting post-program focus groups, the evaluation captured authentic, real-time student perceptions of program acceptability, learning, and engagement within the school food environment. These student-centered outcomes provide valuable insight for refining intervention delivery, enhancing feasibility, and supporting sustainability from the perspective of adolescent participants.

Third, the program was implemented within the natural context of the school lunch period. Delivering brief, theory-informed nutrition education at the point of food service allowed the program to operate within existing school food systems without disrupting instructional time. The real-world implementation strengthens the relevance and transferability of findings for school nutrition professionals and future school-based programming.

Despite these strengths, several limitations should be considered. Post-session survey findings relied on self-reported data, which may be subject to social desirability bias or limited recall, particularly among the younger participants. Nutrition knowledge was assessed qualitatively through brief, open-ended responses collected immediately following each session. While this approach captures students’ immediate recall of session-aligned concepts, it does not provide a standardized or validated measure of nutrition knowledge acquisition, depth of understanding, or retention over time. Responses, therefore, may reflect surface-level recall rather than conceptual comprehension.

Surveys were completed anonymously, and individual students may have contributed responses across multiple sessions. This limits the ability to track individual-level changes, assess cumulative exposure, and stratify responses by other demographics (e.g., age, grade level). Furthermore, students in grades three through eight were grouped within the evaluation, representing a broad developmental range. As such, there may be differences across this age span that influence the depth and the clarity of the qualitative responses that we are unable to account for.

Focus group participants represented a somewhat small subsample of program participants (23%, *n* = 18). While the depth of the qualitative data supports transferability, findings may not be generalizable to urban or suburban school settings or to schools with differing cafeteria infrastructure or food service resources. Students also described perceived changes in behavior outside of school, such as preparing smoothies at home or increasing fruit intake; however, these behaviors were not directly measured within the scope of this study. Importantly, it is unknown whether students had access to key resources at home, such as blenders or smoothie ingredients, which may limit the feasibility of translating school-based experiences into sustained home behaviors.

Finally, this evaluation was not designed to assess long-term behavior change or maintenance of perceived changes. Outcomes reflect short-term experiences, perceptions, and self-reported learning during and immediately following program participation. While the study explored theoretical mechanisms of engagement and agency, it did not quantify the relative contributions of individual SCT or Choice Architecture constructs to longer-term outcomes.

## 5. Conclusions

In conclusion, this mixed-methods evaluation provides a student-centered examination of how adolescents experienced the HEALTHY program when delivered within the school food environment. Findings suggest that students generally perceived the program as acceptable and engaging, demonstrated immediate recall of session-aligned nutrition concepts, and described experiences consistent with selected constructs of SCT and Choice Architecture. The prominence of environmental and social themes highlights the importance of situating nutrition education within the contexts in which food decisions are made, particularly in rural school settings where structural barriers to nutrition security may persist [5,6,7,12,33,34,35].

At the same time, results should be interpreted within the context of the study’s design. Outcomes reflect short-term perceptions and self-reported experiences, and knowledge recall was assessed qualitatively rather than through validated measures. Although some students described behavior changes beyond the school setting, longer-term impacts were not evaluated. Future research may benefit from examining sustained behavioral outcomes, developmental differences across grade levels, and the relative contribution of specific theoretical components. Collectively, these findings contribute preliminary evidence that brief, theory- and school-based nutrition education programming may represent a feasible approach to engaging rural adolescents within existing school food systems.

## Figures and Tables

**Table 1:** *Semi-Structured Focus Group Guide for Assessing Student Experiences with the HEALTHY (Helping Early Adolescents Live Their Healthiest Youth) Program*.

Open-Ended Questions	
1	How would you describe your school lunch experience?
2	Did you notice any changes in your school cafeteria this year? If yes, what were they?
3	How did you feel about the educational smoothie taste tests we had in your school?
4	Did you try any new foods because of the changes in the cafeteria? If yes, what do you think of them?
5	Do you think the educational smoothie taste tests helped you learn more about healthy eating or fruit and vegetable intake? Why or why not?
6	Have you made any changes to what you do at home or school because of this program? If yes, can you share what those changes are?
7	Was there anything about the educational smoothie taste tests that worked well or didn’t work well?
8	What could we do differently to make the educational smoothie taste tests better?
9	Is there anything you think would encourage you or your classmates to eat more fruits?

**Table 2:** *Codebook based on Social Cognitive Theory (SCT) [17] for Deductive Analysis of Student Focus Groups*.

SCT Domain	A Priori Codes	Definitions
Cognitive	Nutrition Knowledge Awareness	Learning new nutrition-related information or awareness related to nutrition behaviors.
	Outcome Expectations	Beliefs about the positive outcomes of healthy eating.
	Perceived Health Benefits	Beliefs about specific positive health impacts of eating healthier foods.
Behavioral	Observational Learning	Observing peers/role models engaging in healthy behaviors and being influenced.
	Positive Behavior Change	Actual behavior changes related to the program.
	Self-Efficacy and Confidence	Expressing confidence in ability to complete new skills/choices.
	Goal-Setting and Self-Regulation	Self-monitoring, goal-setting.
	Sharing or Applying Nutrition Knowledge	Sharing learned knowledge/promoting health to others.
Environmental	Facilitation and Behavioral Capability	Physical/visual cafeteria tools/factors/cues.
	Group Norms and Collective Efficacy	Classmates/school community collectively engaged/supported healthy behaviors.
	Interpersonal Encouragement	Direct encouragement/support from staff/family/peers to engage in healthy behaviors.
	Incentive Motivation	Rewards to initiate behaviors.
	Environmental Barriers	Challenges or obstacles in the school cafeteria.
	Accessibility of Healthy Foods	Ease/frequency of availability in the school cafeteria.

**Notes:** SCT: Social Cognitive Theory.

**Table 3:** *Student-Reported Smoothie Likeability and Willingness to Consume Again by HEALTHY (Helping Early Adolescents Live Their Healthiest Youth) Program Session (N = 360)*.

	Session 1 Smoothie	Session 2 Smoothie	Session 3 Smoothie	Session 4 Smoothie
Likeability Level				
Loved it!	43.0 (43)	48.9 (43)	25.8 (23)	24.1 (20)
It was good.	44.0 (44)	33.0 (29)	31.5 (28)	41.0 (34)
It was okay.	11.0 (11)	12.5 (11)	32.6 (29)	19.3 (16)
Didn’t like it much.	2.0 (2)	5.7 (5)	10.1 (9)	15.7 (13)
Willingness to Consume or Make				
(At Home)				
Affirmative	94.0 (94)	89.8 (79)	80.9 (72)	72.3 (60)
Negative	6.0 (6)	10.2 (9)	19.1 (17)	27.7 (23)
Willingness to Consume				
(At School Lunch)				
Affirmative	97.0 (97)	95.5 (84)	88.8 (79)	79.5 (66)
Negative	3.0 (3)	4.5 (4)	11.2 (10	20.5 (17)

**Notes:** Values are presented as % (*n*). Affirmative responses are considered selected options of “Yes” and “Maybe.” Negative responses are regarded as those who selected “Probably not.” Sample sizes varied across program sessions and are as follows: session 1 (*n* = 100), session 2 (*n* = 88), session 3 (*n* = 89), and session 4 (*n* = 83).

**Table 4:** *Major Themes and Selected Representative Quotes from Open-Ended Survey Responses from Students Participating in the HEALTHY (Helping Early Adolescents Live Their Healthiest Youth) Program (N = 360)*.

Major Themes, % (*n*) Student Agreement	Selected Representative Quotes
Session-Aligned Nutrition Knowledge 53.0% (*n* = 191)	“Berries help you from getting sick, and they have vitamins in them.” (session 1 student) “That mixed berries have vitamin C, I think.” (session 1 student) “The yogurt has protein.” (session 1 student) “Vitamin A and C are good for your skin.” (session 2 student) “You can get hydrated from fruit.” (session 3 student) “How peaches are good for you.” (session 3 student) “That we can make smoothies with foods local to Maine.” (session 4 student)
HEALTHY Program Acceptability 29.0% (*n* = 106)	“It’s good as is.” (session 1 student) “I like the stickers and prizes.” (session 1 student) “Nothing, it was so fun!” (session 2 student) “Nothing, I loved it!“ (session 2 student) “Nothing, it’s the best.” (session 3 student) “It is already fun.” (session 3 student) “IDK, I love your program already.” (session 4 student) “Not a thing!” (session 4 student)
Program Improvement Ideas 38.0% (*n* = 139)	“Bigger smoothie.” (session 1 student) “More banana and peanut butter.” (session 1 student) “More smoothies and flavors.” (session 2 student) “Ring toss to the smoothie you want.” (session 2 student) “No lactose/dairy.” (session 2 student) “If I could make it.” (session 2 student) “More Prizes.” (session 3 student) “Making it with friends.” (session 3 student) “Guessing ingredients.” (session 4 student) “A smoothie bar.” (session 4 student) “Painting.” (session 4 student)
Unclear Knowledge or Improvement Ideas 23.0% (*n* = 83)	“I don’t know.” (session 1—4 students) “???” (session 1—4 students) “I don’t know yet.” (session 2 student) “IDK [I don’t know], but it was super good.” (session 3 student)

**Table 5:** *Representative Quotes by Social Cognitive (SCT) [17] Domain and Construct Identified in Focus Group Discussions with Students Participating in the HEALTHY (Helping Early Adolescents Live Their Healthiest Youth) Program (N = 18)*.

A Priori Codes (%, *n*)	Selected Representative Quotes
**Cognitive Domain**	
Nutrition Knowledge Awareness (44.4%, *n* = 8)	“Yeah, cause like, sometimes your body needs more of this vitamin […], to like, build more strength, and have a healthier immune system, and like to function better. I like learning about that stuff too, to know what’s going to make my athletic performance better.” (school A student) “Sometimes on the SMART goals with it, it tells us what foods are good for you and what foods you should eat more of.” (school B student)
Perceived Health Benefits (33.3%, *n* = 6)	“I learned that umm eating more berries and milk […] tends to be good, help you more.” (school B student) “I also notice people’s moods have started to change as they are trying new things. They are in a better mood because they feel better.” (school B student)
Student Driven Suggestions *^a^* (61.1%, *n* = 11)	“One of the things I thought was kind of um, what is it, not happy about was the serving size. It was really small… I think there should be a size that you can pick.” (school A student) “Let us try to guess what is in the smoothie by tasting it, and if we do you give us a sticker or something.” (school B student)
**Behavioral Domain**	
Positive Behavior Change (33.3%, *n* = 6)	“Like, with the smoothie cards, I still have those in our cookbook at home. That’s what we use them still. And it just opened up, like, a whole new thing in school. It made it more fun.” (school A student) “I feel like the smoothie thing helps a lot with the different varieties of smoothies because you can see which one you like, and you can make it at your house. It’s also helping people eat more healthy and be better with healthy stuff.” (school B student)
Self-Efficacy and Confidence (38.3%, *n* = 7)	“I started making more smoothies at home.” (school B student) “Especially the recipe cards, because I made a bunch of those.” (school A student)
Goal-Setting and Self-Regulation (44.4%, *n* = 8)	“They [SMART goals], they helped a lot by telling you what you should do to stay healthy as much as you.” (school B student) “I think a goal I set for myself […], to eat some fruits and veggies with my meals.” (school B student)
**Environmental Domain**	
Group Norms and Collective Efficacy (55.5%, *n* = 10)	“But I also think some parents aren’t good role models for their kids, like we are not with eating fruits. So maybe if more parents ate fruit, or like if you have older siblings, or eating something like that, I feel like that would be good because then you feel like you want to eat more fruits because they are doing it.” (school B student) “They were really fun. I think we should just have a smoothie bar now. They were so yummy.” (school A student)
Interpersonal Encouragement (33.8%, *n* = 7)	“It’s also uh, I know for a lot of people, I know for me too, it’s easier for me to ask for more things when they’re [cafe staff] nicer.” (school A student) “I just wanna say our school has gotten way better with fruits and vegetables since you guys came.” (school B student)
Incentive Motivation (61.0%, *n* = 11)	“Maybe the colors [of the straws] help us be motivated because we like the colors.” (school B student) I like that it had prizes, so it encouraged us to try new stuff.” (school B student)
Environmental Barriers (33.3%, *n* = 6)	“Well, I mean, I guess, motivation because a lot of people don’t, well, a lot of people don’t have that environment around them to push them to eat that stuff. They are just surrounded by junk food, and bad habits around them, or their parents eat that.” (school A student) “Your parents are healthy, then you will probably just watch them, and grow up and just [be healthy].” (school A student)
Accessibility of Healthy Foods (55.5% *n* = 10)	“Well, yeah, but the smoothies though. Because if kids can’t have access to it [at home], then they can have it at school.” (school A student) “Um I’ve noticed more people have been coming, more people have been eating more fruit, more people have been eating smoothies every time you come, and more stuff, you know.” (school B student)
Social Atmosphere *^a^* (61.0%, *n* = 11)	“Also, lunch is probably my favorite because, umm, I get to sometimes sit with my friends.” (school B student) “It’s gotten better; the lunch ladies have become more social with me.” (school B student)

**Notes:** SCT: Social Cognitive Theory. *^a^* Denotes new emergent codes that appeared outside of a priori codebook.

## Data Availability

Data are available upon reasonable request made to the authors. The data are not publicly available due to privacy and ethical reasons.
